# 3D-printed Model and guide plate for accurate resection of advanced cutaneous squamous cell carcinomas

**DOI:** 10.3389/fsurg.2022.964210

**Published:** 2023-01-06

**Authors:** Weiqian Jiang, Peng Chen, Lei Cui, Li Li, Yan Shao, Dekang Zhang, Lin Xu, Ran Tao, Youbai Chen, Yan Han

**Affiliations:** ^1^ Department of Plastic and Reconstructive Surgery, The First Medical Center, Chinese PLA General Hospital, Beijing, China; ^2^ The Graduate School, The Medical School of Chinese PLA, Beijing, China; ^3^ Department of Oral and Maxillofacial Surgery, The First Medical Center, Chinese PLA General Hospital, Beijing, China; ^4^ Department of Radiology, The First Medical Center, Chinese PLA General Hospital, Beijing, China

**Keywords:** CSCC, MRI, 3D print, tumor model, resection guide plate

## Abstract

**Purpose:**

Advanced cutaneous squamous cell carcinomas (cSCC) can have unclear borders, and simple expanded resection may not only destroy surrounding normal tissues unnecessarily, but can also leave residual tumor cells behind. In this article, we describe a new method for resection and evaluate its accuracy.

**Methods:**

The magnetic resonance imaging (MRI) data of 12 patients with advanced cSCC were reconstructed to obtain three-dimensional (3D) tumor models and guide plates for surgeries. Thirty-eight patients with the same cSCC stage, who underwent expanded resection, were included. The distances between the upper, lower, left and right horizontal margins and tumor pathological boundaries were classified as “positive”, “close” (0–6 mm), “adequate” (6–12 mm) or “excessive” (>12 mm). The positive margin rate and margin distance were compared between the groups.

**Results:**

The 3D tumor models of 12 patients were all successfully reconstructed. The positive rate of 48 surgical margins in the guide plate group was 2.1%, and the proportion of “adequate” margins was 70.8%. A total of 152 margins of 38 patients were included in the extended resection group, for which the positive rate was 13.8%; this was higher than that of the guide plate group (*P* = 0.045). The proportion of “adequate” margins was 27.6%, with group differences seen in the distance distribution (*P* < 0.01).

**Conclusions:**

In surgical resection of advanced cSCC, compared with simple expanded resection, surgical planning using a 3D tumor model and guide plate can reduce the rate of horizontal surgical margins, and the probability of under- or over-resection.

**Clinical Trial Registration**: http://www.chictr.org.cn, Identifier [No. ChiCTR2100050174].

## Graphical abstract

**GRAPHICAL ABSTRACT:**
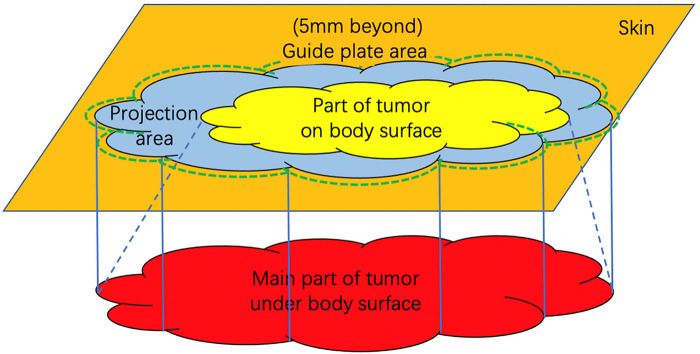
Schematic figure of the relationship between guide plate and tumor. The orange area represents the body surface; the red area represents the maximum range of tumor subcutaneous invasion in MRI; the yellow area represents the visible part of the tumor on the body surface; the blue area represents the projection range of the tumor on body surface and the green dotted line represents the guide plate range to help resection, which is 5 mm beyond the projection boundary.

## Introduction

Cutaneous squamous cell carcinoma (cSCC) is the second most common skin cancer following basal cell carcinoma. The prognosis of primary localized cSCC is overall favorable after complete surgical removal (1). To achieve complete removal, the 2020 National Comprehensive Cancer Network (NCCN) Guideline recommended a 4 mm to 9 mm extended resection of CSCC according to its location, invasion, pathological subtypes, etc. (2–4). However, advanced CSCC (ACSCC) often has a larger size and deeper invasion and is more invasive (5). The recommended extension may be inadequate, leading to a higher risk of recurrence, metastasis, and mortality. Current methods may not accurately determine the surgical margin of ACSCC because the subcutaneous tumor entity is irregular, and often larger than its skin lesion. For example, Mohs microsurgery is infeasible and time-consuming. In addition, the commonly-used traditional intraoperative frozen section has a higher false negative rate in identifying poorly-differentiated, lymphatic and vascular invasive ACSCC. Therefore, it is crucial to develop a method that facilitates accurate and time-saving removal of ACSCC while preserving surrounding normal tissues.

High resolution magnetic resonance imaging (MRI) is useful in evaluating the invasion of soft tissue tumors (6–9). MRI Three-dimensional (3D) reconstruction has been used in neurosurgery and many other specialties to guide tumor resection (10–12), including CSCC (9, 13). For example, Tang showed that CSCC had a mixed signal on T1WI and T2WI, and obvious inhomogeneous enhancement (14). Our previous studies demonstrated that MRI can accurately display the boundaries of tumor body (15). Tumor that invaded peripherally had a high signal and unclear boundary on T1WI. However, the conventional 2D-imaging is unable to accurately reflect the complex 3D structure, reproduce the actual spatial geometry, and offer tactile feedback. As the development of 3D printing technology, 3D-printed models have gained global popularity owing to their individualization, visualization, and tangibility with actual dimensions and spatial geometry. However, no study to date reported the use of MRI-based 3D-printed tumor model and guide plate for accurate resection of ACSCC.

The purpose of this study is to print 3D models and guide plates of ACSCC based on high resolution MRI data to guide surgical resection. The potential benefits for intraoperative margin control were also evaluated.

## Patients and methods

### Study design

After approval by the Ethics Committee of Chinese PLA General Hospital (S2021-662-01) and registration on http://www.chictr.org.cn (ChiCTR2100050174), we conducted a non-randomized controlled trial on patients with ACSCC at the Department of Plastic and Reconstructive Surgery, Chinese PLA General Hospital from October 2018 to May 2021.

### Eligibility

The inclusion criteria were: (1) Patients underwent preoperative biopsy and the lesion was pathologically confirmed as CSCC; (2) Patients received MRI; (3) Patients with ACSCC. ACSCC was defined as the American Joint Committee on Cancer (AJCC) stage ≥T3, i.e., maximum diameter ≥4 cm, minor bone erosion, perineural invasion, or deep invasion as shown by the biopsy and/or imaging (2, 16). Perineural invasion was defined as tumor cells within the nerve sheath of a nerve lying deeper than the dermis or measuring 0.1 mm or larger in caliber, or presenting with clinical or radiographic involvement of named nerves. Deep invasion was defined as invasion beyond the subcutaneous fat or >6 mm (as measured from the granular layer of adjacent normal epidermis to the base of the tumor). The Breslow thickness of the lesion was ≥2 mm and Clark classification was V, i.e., the tumor invasion beyond the subcutaneous fat tissue; (4) Patients agreed to participate in the research and signed informed consent. Exclusion criteria were: (1) Patients without preoperative MRI or biopsy; (2) Contraindications for surgery; (3) Distant metastasis of the ACSCC was confirmed by PET-CT or -MRI; (4) Patients refused to participate in the research. All patients provided written informed consent.

Thirty-eight previous patients with the same stage of cSCC, who underwent conventional extended resection were included as controls. In the 3D group, MRI-based 3D-printed models and guide plates were intraoperatively used to determine the surgical margins. In the control group, surgical margins were decided at least 6-mm away from the visible tumor boundary according to the NCNN/AJCC guidelines (17–19) and at the discretion of the attending surgeon.

### Reconstruction, print, and use of 3d tumor models and guide plates

**Figure 1:**
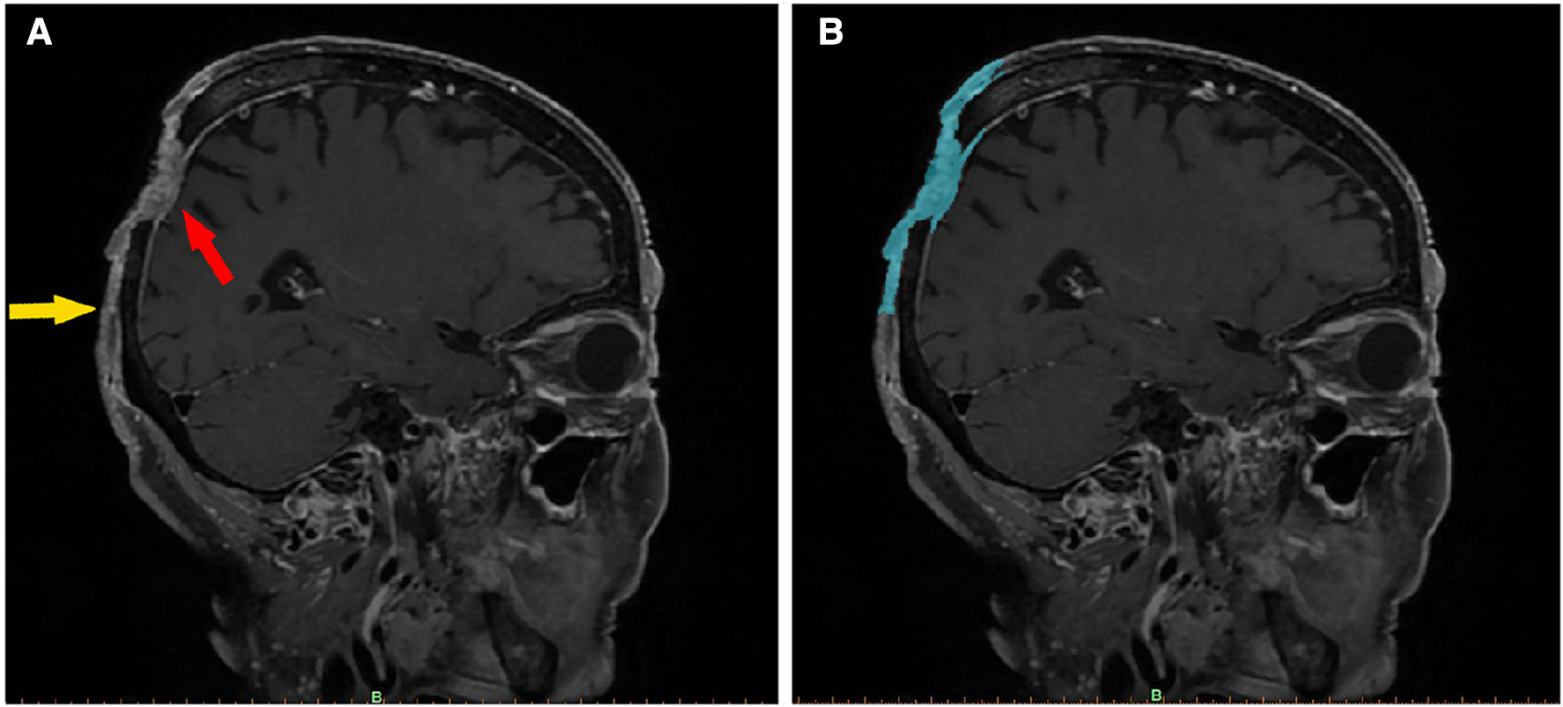
Imaging features of a scalp SCC (enhanced fat-saturated T1WI 3D CUBE sequence). In image (**A**), the red arrow indicates the main part of the tumor, which has an irregular, mixed signal, has invaded the dura mater, and is pressing inward. The yellow arrow marks the boundary of the tumor, the upper part of which is connected to the main part of the tumor. The skin is adhered to the periosteum, and the subcutaneous signal disappears. Image (**B**) shows the same section, segmented using Mimics software. The blue area denotes the imaging range. After being segmented layer by layer, the images were merged to create a 3D tumor model.

**Figure 2:**
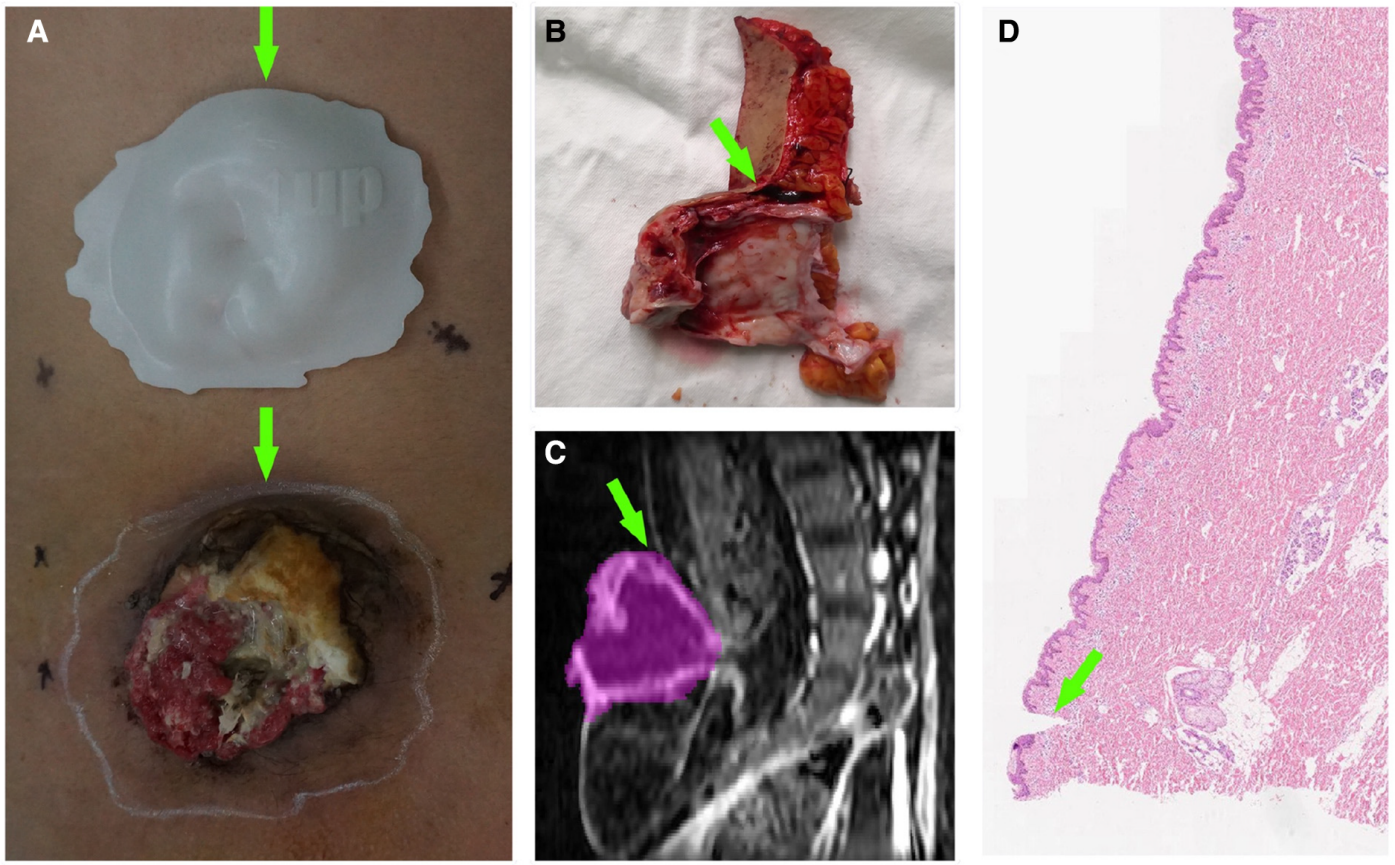
A patient with lumbosacral SCC. (**A**) Comparison between the tumor and guide plate. The white line around the tumor is the imaging boundary (corresponding to the plate); the green arrows denote the 12 o’clock position. (**B**) Margin tissue of the tumor specimen at the 12 o’clock position. The green arrow points toward the scalpel incision (imaging boundary). (**C**) Sagittal MRI image of the tumor at the 12 o’clock position. The purple area denotes the imaging range. (**D**) Pathological section of the margin tissue. The green arrow points toward the scalpel incision, above which the skin tissue is normal.

**Figure 3:**
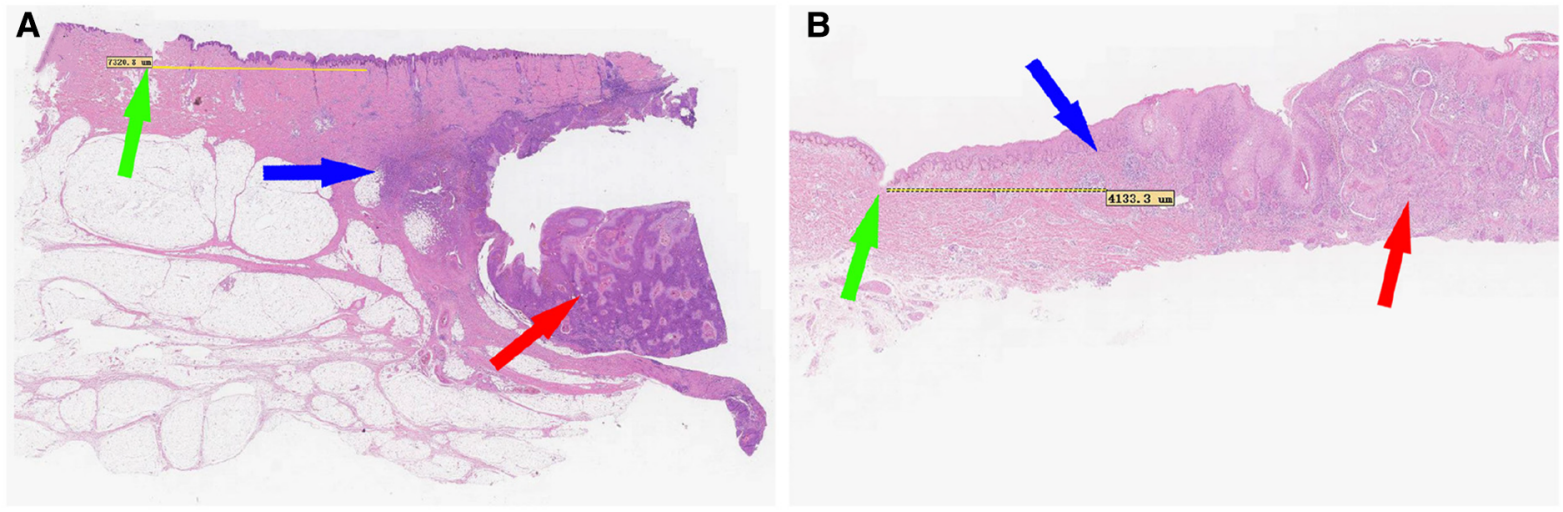
Distance between the tumor edge (according to the guide plate) and scalpel incision. The green arrows indicate the scalpel incisions; the red arrows indicate the main bodies of the cSCC and the blue arrows indicate the pathological boundaries of the tumors. In (**A**), the distance is classified as adequate (7,320.8 μm), and in (**B**), the distance is classified as close (4,133.3 μm).

All patients underwent 3 T MRI scans (MR750; GE, Boston, MA, United States) with 1-mm thickness. The fat-saturated T1-weighted 3D CUBE enhanced sequence was used in the head, face and neck, whereas 3D LAVA sequence was used for ACSCC in the trunk and extremities. In the 3D group, MRI data in the DICOM format were imported to Mimics 20.0 (Materialise NV, Leuven, Belgium). The tumor and surrounding normal soft tissue were distinguished manually on each layer according to their different MRI signal intensity as shown in Figure 1. The 3D models of the tumor and surrounding tissue were reconstructed and exported in STL format. These models were merged and the projected contour of the tumor on the body surface (the imaging boundary) was delineated using Geomagic Design X software (Geomagic, Research Triangle, NC, United States). The surgical margin was decided as 5 mm away from the imaging boundary to design the resection guide plate (20). The data of the 3D model and guide plate were imported into Magics 3D printing software (Materialise). The tumor model and guide plate were printed at the original scale using a photocurable 3D printer (iSLA880; Rapid Tech, Waltham Cross, UK) and were sterilized with ethylene oxide for intraoperative use (Figure 2). ACSCC was removed exactly on the outline of the guide plate. Frozen sections at the 3, 6, 9 and 12 o'clock points were performed to assess the margin. Wound was closed if the frozen section showed that all margins were negative, otherwise a 5-mm extended resection was performed around the positive point until negative pathological result was achieved. In addition, HE staining at the 3, 6, 9 and 12 o'clock points of the removed tumor sample was performed. The distances between the tumor boundary and surgical margin at the four points on HE staining were measured microscopically (Figure 3).

### Medical record retrieval

The medical records of patients with stage T3 or above cSCC, who underwent conventional extended resection from 2015 onward, were collected. The upper, lower, left and right distances between the tumor and surgical edges were recorded based on the pathological results.

### Outcomes

The primary outcome was the positive rate of the surgical margin, which was calculated as the number of positive points divided by the total number of tested points in the intraoperative frozen section. Secondary outcome was the distance from the tumor's pathological margin to the surgical margin at the four points on HE staining. The distance was further categorized as “positive” (0 mm or overlapped), “close” (>0 mm but ≤6 mm), “adequate” (>6 mm but ≤12 mm), and “excessive” (>12 mm) according to previous studies (17–19).

### Statistical analysis

Statistical analysis was performed using SPSS 23.0 software (IBM Corp., Armonk, NY, United States). The two groups were compared in terms of the percentages of positive margins, close distances, excessive distances and adequate distances using the Mann-Whitney U test, and the overall rate of positive resection margins was assessed using the Chi-square test. The level of significance was set at 0.05.

## Results

A total of 12 patients were enrolled in the test group and 38 patients were enrolled in the control group. The characteristics of the patients and tumors are shown in Table 1.

**Table 1:** Patient and tumor characteristics.

Characteristics	Test group	Control group
Males	8 (66.7%)	22 (57.7%)
Females	4 (33.3%)	16 (42.1%)
Age (years)	56.3 ± 19.7	56.3 ± 17.0
**Tumor location**		
Scalp	5 (41.7%)	16 (42.1%)
Face and neck	2 (16.7%)	12 (31.6%)
Trunk	4 (33.3%)	6 (15.8%)
Limb	1 (8.3%)	4 (10.5%)
**Tumor surface area (cm^2^)**		
Maximum	460 (23 × 20)	175 (25 × 7)
Minimum	14 (4 × 3.5)	6 (6 × 1)
Mean ± SD	62.6 ± 126.3	35.58 ± 41.8
**3D tumor model volume (ml)**		
Maximum	1091	–
Minimum	23	–
Mean ± SD	158.2 ± 298.6	–

Data are numbers unless otherwise specified.

**Figure 4:**
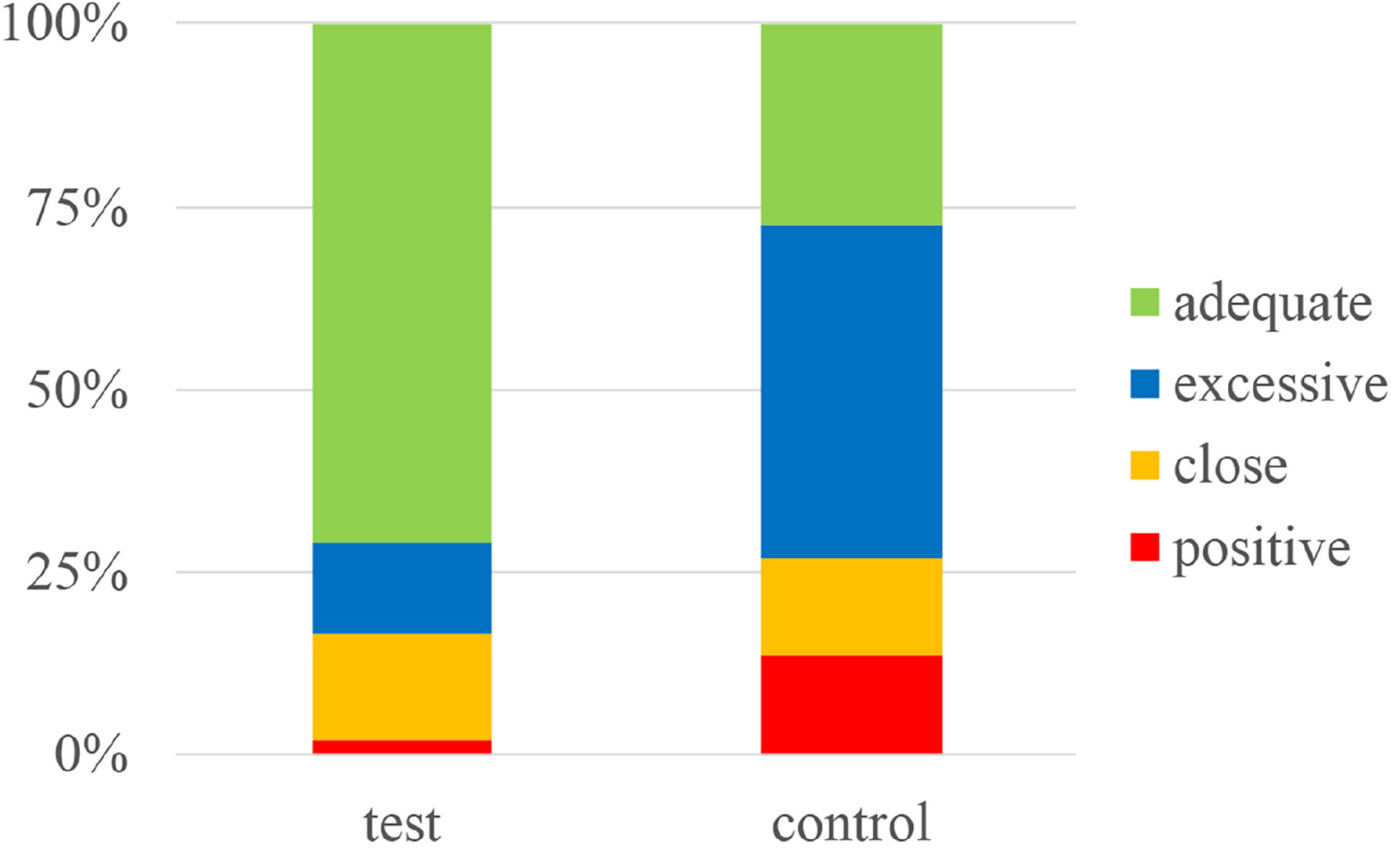
Stacked histogram showing the percentages of positive, close, excessive and adequate margins in the guide plate (test) and conventional extended resection (control) groups.

In the test group, all the 3D tumor models were reconstructed successfully, with an average volume of 158.2 ± 298.5 ml, and 48 pathological sections were made. In the control group, 152 margin distances were included. The typical measurements are shown in Figure 3, and the results are displayed in Table 2 and Figure 4.

**Table 2:** Positive margin rate and margin classifications in the guide plate (test) and conventional extended resection (control) groups.

Outcome	Test	Control	*P*-value
Positive (count)	1	21	0.045
Negative (count)	47	131	
Positive	2.1%	13.8%	<0.01
Close (>0, ≤6 mm)	14.6%	13.2%	
Excessive (>12 mm)	12.5%	45.4%	
Adequate (>6, ≤12 mm)	70.8%	27.6%	

The two groups were not only significantly different in terms of the overall positive resection margin rate (*p* = 0.045), but also in the percentages of positive margins, close distances, excessive and adequate distances (*p* < 0.01). These results showed that using 3D tumor models and resection guide plates to guide surgical resection in cases of cSCC can efficiently lower the rate of positive margins, and keep the distance between the tumor and resection margins within an appropriate range.

### Typical case

**Figure 5:**
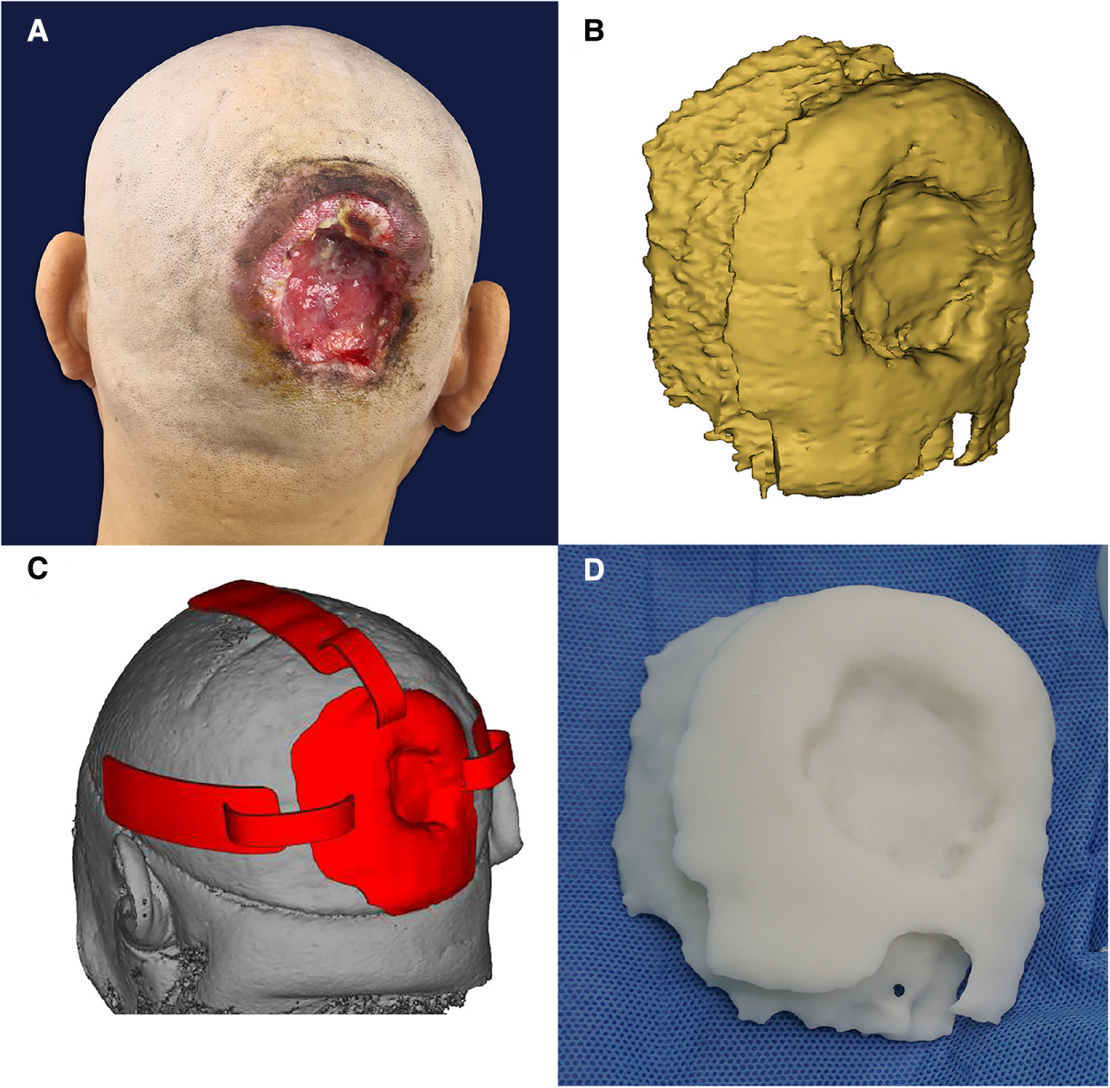
(**A**) Before the operation. (**B**) Three-dimensional digital model of the tumor under reconstruction. (**C**) Resection guide plate and body surface simulation. (**D**) The 3D-printed tumor model.

**Figure 6:**
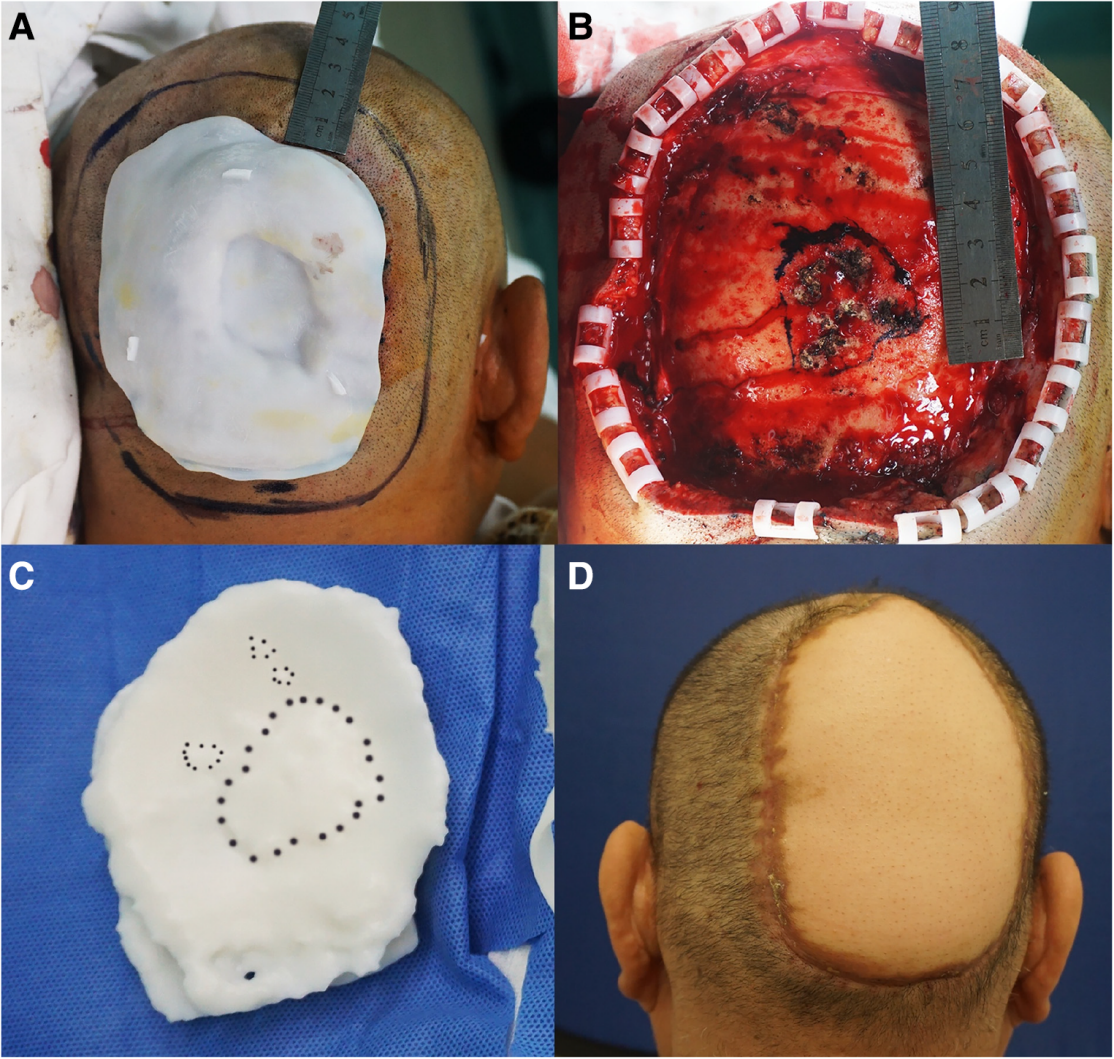
(**A**) The incision line was 2 cm beyond the plate. (**B**) Residual wound after tumor resection. The extent of methylthionine chloride staining of the invaded area of the skull. (**C**) Methylthionine chloride stained the “bulge area” of the model, which corresponded to the area of skull invasion. (**D**) The wound was treated by free skin flap transplantation.

**Figure 7:**
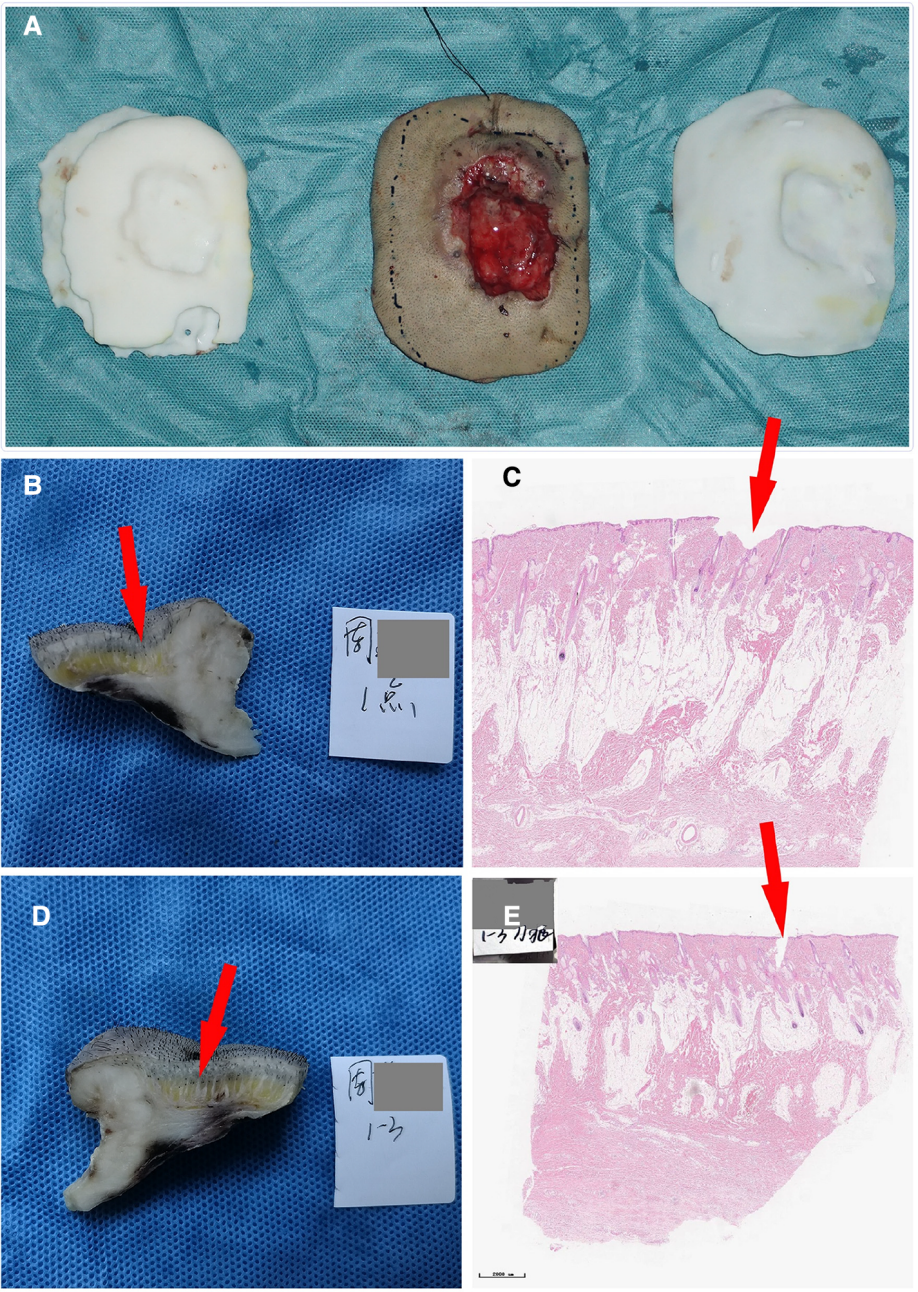
(**A**) Comparison of the tumor specimen, 3D model and guide plate. (**B**) The sample was collected at the 1 o‘clock position; the red arrow indicates the imaging boundary. (**C**) Pathological section of the specimen. (**D**) The sample was collected at the 3 o‘clock position. (**E**) Another image of the pathological section of the specimen. The neoplastic tissues are within the imaging boundary.

The patient was a 46-year-old male with a 20-year history of scalp SCC (moderately to poorly differentiated squamous cell carcinoma). Physical examination revealed a 5-cm crater-like uplift on the occiput, with a circular ulcer in the center that extended into the periosteum, covered by pus and yellowish liquid. The boundary of the uplift was clear, and the surrounding tissue was slightly red and swollen. The enlarged lymph nodes in the mandible and neck were tender on palpation (Figures 5–7).

## Discussion

For stage T3 and above cSCC cases, complete resection is still one of the most important treatment options recommended by current guidelines (2, 18, 19). These tumors have a large surface area and high capacity for subcutaneous invasion. If the extent of conventional resection is insufficient, there is a higher chance of residual recurrence. By contrast, if the extent is large, surrounding healthy tissues can easily be damaged. Additionally, Mohs surgery is no longer recommended as the optimal strategy for this type of high-risk cSCC, due to the high time and labor demands and inaccuracy of frozen sections (4). Therefore, we used 3D tumor models and a resection guide plate, designed based on MRI data, to assist tumor resection and verify that this method can successfully control resection boundaries.

### Reconstructing A 3d tumor model from MRI data

Clinically, MRI is a routine preoperative procedure for soft tissue cutaneous tumors, and is helpful to assess tumor infiltration and metastasis (7). Compared with CT and ultrasound, among other imaging modalities, MRI is preferred for soft tissue imaging; it has a higher resolution, so can be used to evaluate whether the tumor has infiltrated into fat or muscle layers (7, 21, 22). Many studies have used MRI for the diagnosis of cSCC (9, 13). For example, Tang showed that cSCC had a mixed signal on T1WI and T2WI, and obvious inhomogeneous enhancement (14). MRI is also considered the most sensitive imaging modality to evaluate tumor invasion into or along nerves (23). Our previous studies demonstrated that MRI can accurately display the boundaries of the main tumor body. The part of the tumor that breaks through or infiltrates distally has a long signal and unclear boundary on T1WI (15).

As MRI images are 2D, they cannot be used to directly determine the true size of a tumor, or its relationship with surrounding tissues. Therefore, we attempted to reconstruct 3D tumor models from MRI data, by converting the 2D images into 3D models. This technique allows for accurate assessment of tumor size, depth of invasion, subcutaneous invasion area, expected resection area, and the area of skin or flap grafts needed to repair the wound (24, 25). In addition, during the process of tumor reconstruction, adjacent vessels can also be assessed, which helps avoid injury of these vessels during resection, and facilitates planning of the skin flap and identification of perforator vessels to anastomose free skin flaps. By helping doctors design surgical plans, operation time and intraoperative bleeding can be reduced, and good communication between doctors and patients promoted.

High-resolution MRI images provide a basis for reconstruction. In this study, enhanced 3D CUBE and 3D LAVA sequences were adopted, and the thinnest layer was 1 mm. Image segmentation determines the accuracy of the models; we combined automatic and manual segmentation, as well as 2D and 3D segmentation. Automatic segmentation can identify the suspicious area based on the length of the signal, and manual segmentation allows the suspicious area to be further delineated according to anatomical relationships and imaging characteristics. Two-dimensional segmentation is used to assess suspicious single-plane regions. Through continuous multi-plane image reading, suspicious regions can be more fully assessed, while 3D voxel editing allows us to obtain a more complete 3D tumor model.

### Application of the tumor resection guide plate

After reconstruction, it is necessary to accurately match the 3D tumor model to the actual lesion. We tried to use a hand drawing process, body surface projection, augmented reality technology and other methods (26). However, due to the mobility and deformation of soft tissue, these methods do not allow the model and tumor to be accurately matched. Thus, we ultimately determined the tumor boundaries using a 3D printing tumor resection guide plate.

The guide plate is convenient for the following reasons. First, reconstruction of the guide plate is relatively simple. Based on the 3D tumor model and curvature of the surgical site, the position of the tumor on the body surface can be easily reconstructed. Its size, shape and length can be fixed. Second, the guide plate can be applied to irregularly shaped parts, such as the scalp, calf and nodular lesions, for which projection and drawing are not suitable. The curvature of the guide plate is designed according to the shape of the body surface, so that it can fit closely the tumor and surrounding skin. Third, the imaging boundary is accurately delineated. The guide plate markers can be matched with the body surface reference points, and the guide plate is tightly attached to the body surface to prevent displacement. In addition, the variability in shape of the guide plate is small, even when disinfected by ethylene oxide in preparation for use during the operation.

To overcome the problem of unclear tumor boundaries on MRI and ensure complete tumor resection, we enlarged the guide plate by 5 mm beyond the scope of the 3D tumor model (20). The margin of cSCC has the character of satellite lesions, so the negative margin under the microscope may still have residual tumor cells. Sepehripour et al. previously reported a histological margin of cSCC in non-recurrent patients of 5.2 ± 2.86 mm, compared with 4.77 ± 2.84 mm in cases of recurrence (27). Therefore, in addition to the rate of margins, we also counted the distances between the incisal margin and the pathological boundary of the tumor and defined the distance of 6–12 mm as adequate, while 0–6 mm was considered close, and > 12 mm excessive (17–19). This study demonstrated that, compared with conventional extended resection, our method yielded more favorable margins. Most (97.9%) horizontal incision margins were negative, and in the majority of cases (70.8%), the distance from tumor to the incisal margin was adequate (6–12 mm). Our method can facilitate complete tumor resection, while largely preserving healthy tissue to prevent undue trauma.

### Limitations of the study

The main limitation of the present study was the potential for sampling error. For every specimen, only four lateral checkpoints (upper, lower, left and right) were pathologically examined and compared with previous cases. This is partly due to the routine intraoperative frozen pathological examination of these four points to assess the presence or absence of residual tumor cells before repairing the wound. Besides, these four points are also the sites of routine postoperative pathological examination and the results can be accessed from the medical records. Therefore, we compared the pathological results of these four points between the guide plate group and previous extended resection group.

In addition, cSCC tumors typically exhibit deep infiltration. Our 3D tumor model can assess tumor invasion and reveal the breakthrough location. However, as the soft tissue is deformed during the incision, the location of the tumor suggested by the model may be obstructed. Therefore, our method needs to be further improved to accurately locate the tumor base. Fortunately, Tan et al. showed that, most cSCC excision failures involved the lateral margin (62%) rather than the deep margin (3.4%) (28). Therefore, our method still has great clinical potential.

Follow-up revealed local recurrence in some of our patients with negative margins, possibly due to vascular infiltration, perineural invasion, or satellite or in-transit metastases (19, 29–32). According to NCCN guidelines and the AJCC staging manual (2, 33, 34), all of our patients with local recurrence had high-risk (stage IV) tumors with an obvious tendency toward subclinical metastasis, such that the recurrence rate remained high after complete surgical resection of the lesions. In one typical case using the guide plate, the imaging extended far beyond the visual range, and postoperative pathology confirmed that although the surgical margin was negative, the surrounding vasculature had been infiltrated. In this patient, the tumor recurred after >10 months postoperatively. This also suggests that, for advanced cSCC, surgical resection should be combined with other treatment modalities to increase the likelihood of remission (16, 19).

## Conclusion

This study demonstrated significant benefits of our 3D tumor model and tumor resection guide plate for accurate resection of cSCC tumors. The method facilitated the delineation of margins and guided resection. Further improvements and large-scale validation will be the targets of future studies.

## Data Availability

The original contributions presented in the study are included in the article/Supplementary Material, further inquiries for the original contributions in the study can be directed the corresponding authors.
